# Is diabetic retinopathy affected by diabetes type? A retrospective study using electronic medical record data from patients with latent autoimmune diabetes in adults, type 1 diabetes, and type 2 diabetes

**DOI:** 10.1007/s00592-021-01748-0

**Published:** 2021-06-13

**Authors:** Wanyue Li, Zifang Cheng, Yanan Song, Yifan Fang, Ming Yang, Maonian Zhang

**Affiliations:** 1 Medical School of Chinese PLA, Beijing, China; 2 The Northern Medical District of Chinese PLA General Hospital, Beijing, China; 3 Medical Big Data Research Center, Medical Innovation Research Division of Chinese People’s Liberation Army General Hospital, Beijing, China; 4 Department of Ophthalmology, Chinese PLA General Hospital, Beijing, China

**Keywords:** Diabetic retinopathy, Diabetic nephropathy, Latent autoimmune diabetes in adults, Type 2 diabetes mellitus, Type 1 diabetes mellitus, Influencing factors

## Abstract

**Aims:**

To determine whether the occurrence of diabetic retinopathy (DR) and its related factors are affected by diabetes type (latent autoimmune diabetes in adults [LADA], type 1 diabetes mellitus [T1DM], type 2 diabetes mellitus [T2DM]).

**Methods:**

LADA patients were matched for age (± 2 years) and sex to T1DM (1:1) and T2DM (1:2) patients. Retrieved variables included demographic characteristics, diabetes history, laboratory test findings, and history of DR screening, etc. Multiple logistic regression analysis was applied to identify influencing factors of DR. A decision tree was used to explore interactions between diabetes type and other influencing factors of DR.

**Results:**

We included 110 LADA, 101 T1DM, and 220 T2DM patients. DR prevalence was 26.4% in LADA patients, lower than that in T1DM (50.5%) and T2DM (47.7%) patients (*P* < 0.001). Logistic regression analysis demonstrated that diabetes duration (OR = 1.15, 95% CI: 1.1–1.26, *P* < 0.001) and diabetic nephropathy (DN) (OR = 42.39, 95% CI: 10.88–165.11, *P* < 0.001) were independent risk factors for DR, and regular DR screening (OR = 0.33, 95% CI: 0.16–0.69, *P* = 0.003) was an independent protective factor. Decision tree analysis showed that in patients without DN with a diabetes duration of at least 10.5 years, T1DM and LADA patients had a higher incidence of DR than T2DM patients (72.7% vs. 55.1%).

**Conclusions:**

The prevalence of DR in diabetes patients was affected by diabetes duration, DN occurrence, and regular DR screening. Diabetes type indirectly affects DR occurrence through its interaction with diabetes duration and DN. Correct LADA diagnosis is necessary, and DR screening needs to be well-implemented.

**Supplementary Information:**

The online version contains supplementary material available at 10.1007/s00592-021-01748-0.

## Introduction

Latent autoimmune diabetes in adults (LADA) is a subtype of type 1 diabetes mellitus (T1DM) [1, 2]. Patients with LADA do not initially require insulin and have the same clinical characteristics as type 2 diabetes mellitus (T2DM) patients at diagnosis. Within a few years, autoimmune imbalance leads to progressive pancreatic *β*-cell dysfunction and insulin dependency [3]. An estimated 4–14% of LADA patients are initially diagnosed with T2DM [4]. Compared with T1DM, LADA is not uncommon.

Diabetic retinopathy (DR) is a leading cause of vision impairment and blindness in the working-age population globally [5, 6]. Although enormous studies have focused on the epidemiology and risk factors of DR, data on DR in patients with LADA are limited. Only a few studies have introduced the clinical characteristics of DR in LADA patients, and most of them have only focused on its prevalence. For instance, in a comparison of the prevalence and incidence of chronic complications between LADA and T2DM patients, Myhill et al. [7] found that there was a similar prevalence and incidence of retinopathy in the two groups (*P* = 0.22 and 0.64, respectively). In another study aimed at analyzing the relationship between glycaemic variability and DR, researchers reported that no metrics related to glycaemic variability were identified as independent risk factors of DR (standard deviation: *P* = 0.175; coefficient of variation: *P* = 0.769; mean amplitude of glycaemic excursions: *P* = 0.388) in LADA patients [8]; however, other risk factors were not examined. In a study by Park et al. [9] that recruited 432 newly diagnosed diabetes mellitus patients, six patients diagnosed with LADA demonstrated proliferative diabetic retinopathy (PDR) as an initial sign. This suggests that retinopathy may develop rapidly in LADA patients.

Therefore, to better manage LADA patients, this study was designed to determine whether the occurrence of DR and its related factors are affected by diabetes type (T1DM, LADA, and T2DM).

## Materials and method

### Data collection

In this study, the clinical data of patients diagnosed with LADA were extracted from the Chinese PLA General Hospital electronic medical record system from 1 January 2013 to 31 October 2020. Age- (± 2 years) and sex-matched patients with T1DM and T2DM were randomly selected in a 1:1 and 2:1 ratio, respectively, in the same period.

#### Inclusion and exclusion criteria

Included patients were either hospitalized in ophthalmology or had received ophthalmic consultation. Patients with missing data or with cataract, keratitis, corneal speckles, and other eye diseases that affect fundus examination were excluded from the study.

#### Data extraction criteria

The first record of measurement upon admission for each variable was extracted. Diagnostic information of the diseases was extracted from the discharge diagnosis records. Retrieved variables included demographic characteristics (sex and age), history of diabetes (diabetes typing, duration, and number of episodes of ketoacidosis), diabetes complications (diabetic nephropathy, neuropathy, retinopathy, and coronary heart disease), hypertension, laboratory parameters (fasting blood glucose, postprandial blood glucose, glycosylated hemoglobin [HbA1c], triglycerides, total cholesterol, high-density lipoproteins, low-density lipoproteins, urea, serum creatinine, and urinary microalbumin/creatinine), physical indicators (weight, height, and systolic and diastolic blood pressure), DR screening history (absence of DR screening, regular DR screening, and number of years until the first fundus examination after diabetes diagnosis), and smoking history. Body mass index (BMI) was calculated as the weight (kg) divided by height in meters squared (m^2^).

#### Diagnostic criteria

The diagnostic criteria for LADA in our hospital are as follows: > 30 years of age, positivity for glutamic acid decarboxylase autoantibodies (GADA), and no requirement of insulin within at least 6 months after diagnosis. The diagnostic criteria for T1DM and T2DM followed those set forth by the 2003 American Diabetes Association (ADA) guidelines [10]. DR was diagnosed according to the International Clinical Diabetic Retinopathy Severity Scale [11] by using color fundus photography and indirect ophthalmoscopy when the pupils were dilated. Fundus examination, image reading, and diagnosis were performed by at least two experienced ophthalmologists for each patient, whether directly admitted to the ophthalmology department or for ophthalmic consultation in other departments.

This retrospective study was approved by the Chinese PLA General Hospital clinical research ethics committee (No. S2019-326-02, February 25, 2020) and adhered to the tenets of the Declaration of Helsinki.

### Statistical analyses

In this study, IBM SPSS, version 23.0 (IBM Corp., Armonk, NY, USA) was used for statistical analyses. Categorical variables were expressed as numbers and percentages. Continuous variables were expressed as means with standard deviations. The Student’s *t* test and analysis of variance (ANOVA) were performed for continuous variables to assess the statistical significance of differences between groups. The chi-square test was used to compare categorical variables. Multiple logistic regression analysis was applied to the whole dataset to identify the influencing factors of DR. The variables included in the logistic regression analysis were determined based on the *P* value in the univariate analysis and applying collinearity diagnostics. The Hosmer–Lemeshow test was used to assess the goodness of fit of the model. A decision tree was used to explore the interactions between diabetes types and other influencing factors with respect to DR. A value of *P* < 0.05 was considered statistically significant.

## Results

### Baseline analysis of three types of diabetes

A total of 110 LADA, 101 T1DM, and 220 T2DM patients were enrolled in the analysis. The clinical characteristics and laboratory test results in each group are shown in Table 1. Patients with LADA, T1DM, and T2DM were similar in terms of age, sex, smoking history, and levels of total cholesterol, urea, serum creatinine, fasting blood glucose, and postprandial blood glucose. Diabetes duration was shorter in LADA patients than in those with T1DM and T2DM (6.01 ± 5.65 vs. 10.70 ± 9.55 vs. 8.97 ± 7.15, *P* < 0.001) when each group was matched by age.

**Table 1:** Clinical characteristics and laboratory tests of patients with T1DM, T2DM and LADA

Variables	Type 1 diabetes(*n* = 101)	Type2 diabetes(*n* = 220)	LADA(*n* = 110)	*P*
Male, *n* (%)	60(59.4%)	130(59.1%)	65(59.1%)	0.998
Age, years	45.64 ± 11.27	46.50 ± 10.93	46.11 ± 11.03	0.81
Duration of diabetes, years	10.70 ± 9.55a	8.97 ± 7.15a	6.01 ± 5.65b	< 0.001**
BMI, kg/m^2^	22.18 ± 2.88a	26.32 ± 3.90b	21.60 ± 3.03a	< 0.001**
Systolic blood pressure, mmHg	127.16 ± 19.73a	136.60 ± 22.40b	121.40 ± 19.51a	< 0.001**
Diastolic blood pressure, mmHg	74.03 ± 11.51a	81.06 ± 14.34b	73.65 ± 11.28a	< 0.001**
Total cholesterol, mmol/L	4.19 ± 0.98	4.49 ± 1.34	4.25 ± 1.09	0.068
Triglyceride, mmol/L	1.17 ± 0.72a	2.34 ± 2.44b	1.04 ± 0.92a	< 0.001**
High-density lipoproteins, mmol/L	1.44 ± 0.47a	1.07 ± 0.29b	1.47 ± 0.45a	< 0.001**
Low-density lipoproteins, mmol/L	2.50 ± 0.85a	2.83 ± 0.97b	2.56 ± 0.99a	0.005**
Urea, mmol/L	7.41 ± 6.84	7.26 ± 5.86	5.90 ± 3.66	0.08
Serum creatinine, μmol/L	137.67 ± 202.21	128.95 ± 195.60	85.06 ± 127.21	0.065
Urinary microalbumin/creatinine	181.75 ± 373.09a	162.00 ± 299.94a	36.93 ± 103.24b	< 0.001**
Fasting blood glucose, mmol/L	9.66 ± 3.92	8.89 ± 3.14	9.43 ± 3.79	0.144
Postprandial blood glucose, mmol/L	14.40 ± 5.33	13.07 ± 4.40	13.74 ± 5.40	0.073
HbA1c, %	8.79 ± 1.95a	8.50 ± 2.26a	9.52 ± 2.31b	< 0.001**
Ketoacidosis, *n* (%)				< 0.001**
Never	35(34.7%) a	188(85.5%) b	52(47.3%) a	
1 time	48(47.5%) a	27(12.3%) b	48(43.6%) a	
More than 1 time	18(17.8%) a	5(2.3%) b	10(9.1%) a	
Smoking, *n* (%)	37(36.6%)	91(41.4%)	39(35.5%)	0.515
Regular DR screening, *n* (%)	30(29.7%) a	32(14.5%) b	42(38.2%) a	< 0.001**
Never DR screening, *n* (%)	46(45.5%)	116(52.7%)	57(51.8%)	0.475
First DR screening, years	7.97 ± 7.77a	6.91 ± 6.77a	4.53 ± 4.94b	< 0.001**
Hypertension, *n* (%)	31(30.7%) a	122(55.5%) b	20(18.2%) a	< 0.001**
Coronary heart disease, *n* (%)	17(16.8%) a	14(6.4%) b	14(12.7%) a, b	0.011*
Diabetic nephropathy, *n* (%)	21(20.8%) a, b	54(24.5%) b	12(10.9%) a	0.014*
Diabetic neuropathy, *n* (%)	31(30.7%) a, b	93(42.3%) b	26(23.6%) a	0.002**
Diabetic retinopathy, *n* (%)	51(50.5%) a	105(47.7%) a	29(26.4%) b	< 0.001**

Values are expressed as the mean ± SD or number (percentages).

T1DM, type 1 diabetes mellitus; LADA, latent autoimmune diabetes in adults; T2DM, type 2 diabetes mellitus; BMI body mass index; HbA1c, glycosylated hemoglobin; DR diabetic retinopathy.

*P < 0.05 for multi-group variance analysis or chi-square test; **P < 0.01 for multi-group variance analysis or chi-square test; a/b, for each variable, there was no significant difference between the groups marked with the same letter

Patients with T2DM had higher BMI scores; systolic blood pressure; diastolic blood pressure; and levels of triglycerides, low-density lipoprotein, and lower high-density lipoprotein than LADA and T1DM patients. There were no significant differences in those variables between the LADA and T1DM groups. Patients with T2DM were less likely to develop ketoacidosis repeatedly over the course of the disease (T1DM 17.8%, T2DM 2.3%, LADA 9.1%; *P* < 0.001). The prevalence of DR was 26.4% in LADA patients, which was lower than that in T1DM and T2DM patients (50.5% and 47.7%, respectively; *P* < 0.001).

Only 14.5% of T2DM patients underwent regular DR screening every year; this proportion was significantly lower than those in T1DM and LADA patients (29.7% and 38.2%, respectively; *P* < 0.001). The first DR screening was performed earlier in LADA patients than in T1DM and T2DM patients. Almost half of the diabetes patients never underwent a fundus examination after diagnosis (T1DM 45.5%, T2DM 52.7%, LADA 51.8%), which means that they received their first fundus examination during this hospitalization.

### Influencing factors of diabetic retinopathy

All diabetes patients were divided into two subgroups: the DR group and the non-DR (NDR) group. According to the results of univariate analysis, patients with DR had a longer diabetes duration (13.09 ± 7.75 years vs. 5.25 ± 5.53 years, *P* < 0.001), higher fasting blood glucose levels (9.41 ± 3.80 mmol/L vs. 9.061 ± 3.27 mmol/L, *P* = 0.047), higher BMI (24.35 ± 3.63 kg/m^2^ vs. 23.99 ± 4.45 kg/m^2^, *P* = 0.033), higher low-density lipoprotein levels (2.80 ± 1.07 mmol/L vs. 2.60 ± 0.87 mmol/L, *P* = 0.007), higher urinary microalbumin/creatinine levels (266.95 ± 373.69 mg/g vs. 35.26 ± 140.81 mg/g, *P* < 0.001), and a lower proportion of regular DR screening (10.3% vs. 34.6%, *P* < 0.001) than those without DR. Diabetic nephropathy and neuropathy were also more common in patients with DR than in those without (45.4% vs. 1.2%, *P* < 0.001 and 45.4% vs. 26.8%, *P* < 0.001, respectively). The univariate analysis results are shown in Table 2.

**Table 2:** Results of univariate analysis

Variables	NDR(*n* = 246)	DR(*n* = 185)	*P*
Male, *n* (%)	155(63.0%)	100(54.1%)	0.061
Age, years	44.15 ± 11.23	48.92 ± 10.13	0.09
Duration of diabetes, years	5.25 ± 5.53	13.09 ± 7.75	< 0.001**
BMI, kg/m^2^	23.99 ± 4.45	24.35 ± 3.63	0.033*
Systolic blood pressure, mmHg	124.77 ± 20.64	138.14 ± 21.56	0.288
Diastolic blood pressure, mmHg	76.25 ± 13.45	79.22 ± 13.30	0.354
Total cholesterol, mmol/L	4.27 ± 1.06	4.47 ± 1.37	0.068
Triglyceride, mmol/L	1.69 ± 2.02	1.80 ± 1.83	0.56
High-density lipoproteins, mmol/L	1.28 ± 0.46	1.24 ± 0.38	0.133
Low-density lipoproteins, mmol/L	2.60 ± 0.87	2.80 ± 1.07	0.007**
Urea, mmol/L	5.28 ± 2.31	9.17 ± 7.70	< 0.001**
Serum creatinine, μmol/L	72.27 ± 55.41	182.99 ± 259.27	< 0.001**
Urinary microalbumin/creatinine, mg/g	35.26 ± 140.81	266.95 ± 373.69	< 0.001*
Fasting blood glucose, mmol/L	9.061 ± 3.27	9.41 ± 3.80	0.047*
Postprandial blood glucose, mmol/L	13.70 ± 5.04	13.37 ± 4.75	0.432
HbA1c, %	9.05 ± 2.22	8.53 ± 2.23	0.517
Smoking, *n* (%)	103(41.9%)	64(34.6%)	0.125
Regular DR screening, *n* (%)	85(34.6%)	19(10.3%)	< 0.001**
Never DR screening, *n* (%)	135(54.9%)	84(45.4%)	0.052
First DR screening, years	3.67 ± 4.31	10.39 ± 7.41	< 0.001**
Hypertension, *n* (%)	71(28.9%)	102(55.1%)	< 0.001**
Coronary heart disease, *n* (%)	23(9.3%)	22(11.9%)	0.393
Diabetic nephropathy, *n* (%)	3(1.2%)	84(45.4%)	< 0.001**
Diabetic neuropathy, *n* (%)	66(26.8%)	84(45.4%)	< 0.001**

Values are expressed as the mean ± SD or number (percentages).

BMI body mass index; HbA1c, glycosylated hemoglobin; DR diabetic retinopathy.

*P < 0.05 for *T* test or chi-square test; **P < 0.01 for *T* test or chi-square test

Logistic regression analysis was performed on all diabetes patients to determine the influencing factors of DR. According to the results of univariate analysis (variables with *P* values < 0.05) and clinical experience, 10 variables were selected, including diabetes duration, BMI, fasting blood glucose, low-density lipoprotein, urinary microalbumin/creatinine, hypertension, regular DR screening, diabetic nephropathy, neuropathy, and type of diabetes. Considering that it can reflect renal function sensitively, urinary microalbumin/creatinine was chosen as the representative of renal function in laboratory tests. Before logistic regression analysis, collinearity diagnostics were performed. As shown in Online Resource 1, there was no collinearity among the 10 variables to be included in the logistic regression analysis.

The associations between DR and the above 10 influencing factors in diabetes patients were assessed using multivariable binary logistic regression, and the calculated odd ratios (ORs) are presented in Table 3. Diabetes duration (OR = 1.15, 95% confidence interval [CI]: 1.1–1.26, *P* < 0.001) and diabetic nephropathy (OR = 42.39, 95% CI: 10.88–165.11, *P* < 0.001) were observed to be independent risk factors for DR, and regular DR screening (OR = 0.33, 95% CI: 0.16–0.69, *P* = 0.003) was an independent protective factor. The Hosmer–Lemeshow test showed that the model seemed to fit well (*P* = 0.178).

**Table 3:** Results of multivariable logistic regression analysis

Variables	OR (95%CI)	*P*
Type of diabetes		0.323
T2DM	0.64(0.30,1.36)	0.246
LADA	0.57(0.26,1.26)	0.166
Duration of diabetes	1.15(1.10,1.21)	0.000**
Hypertension (1)	0.65(0.34,1.22)	0.176
BMI	1.03(0.95,1.11)	0.507
Fasting blood glucose	1.06(0.98,1.16)	0.137
Low-density lipoproteins	1.30(0.97,1.75)	0.084
Diabetic nephropathy (1)	42.39(10.88,165.11)	0.000**
Peripheral neuropathy (1)	1.60(0.92,2.80)	0.099
Regular DR screening (1)	0.33(0.16,0.69)	0.003**
Urinary microalbumin/creatinine	1.00(1.00,1.00)	0.136

Hosmer–Lemeshow test: *P* = 0.178

T1DM, type 1 diabetes mellitus; LADA, latent autoimmune diabetes in adults; T2DM, type 2 diabetes mellitus; BMI body mass index; DR diabetic retinopathy; OR, odd ratio; CI, confidence interval.

*P < 0.05; **P < 0.01

### Influence of diabetes type on the occurrence of diabetic retinopathy

**Fig. 1:**
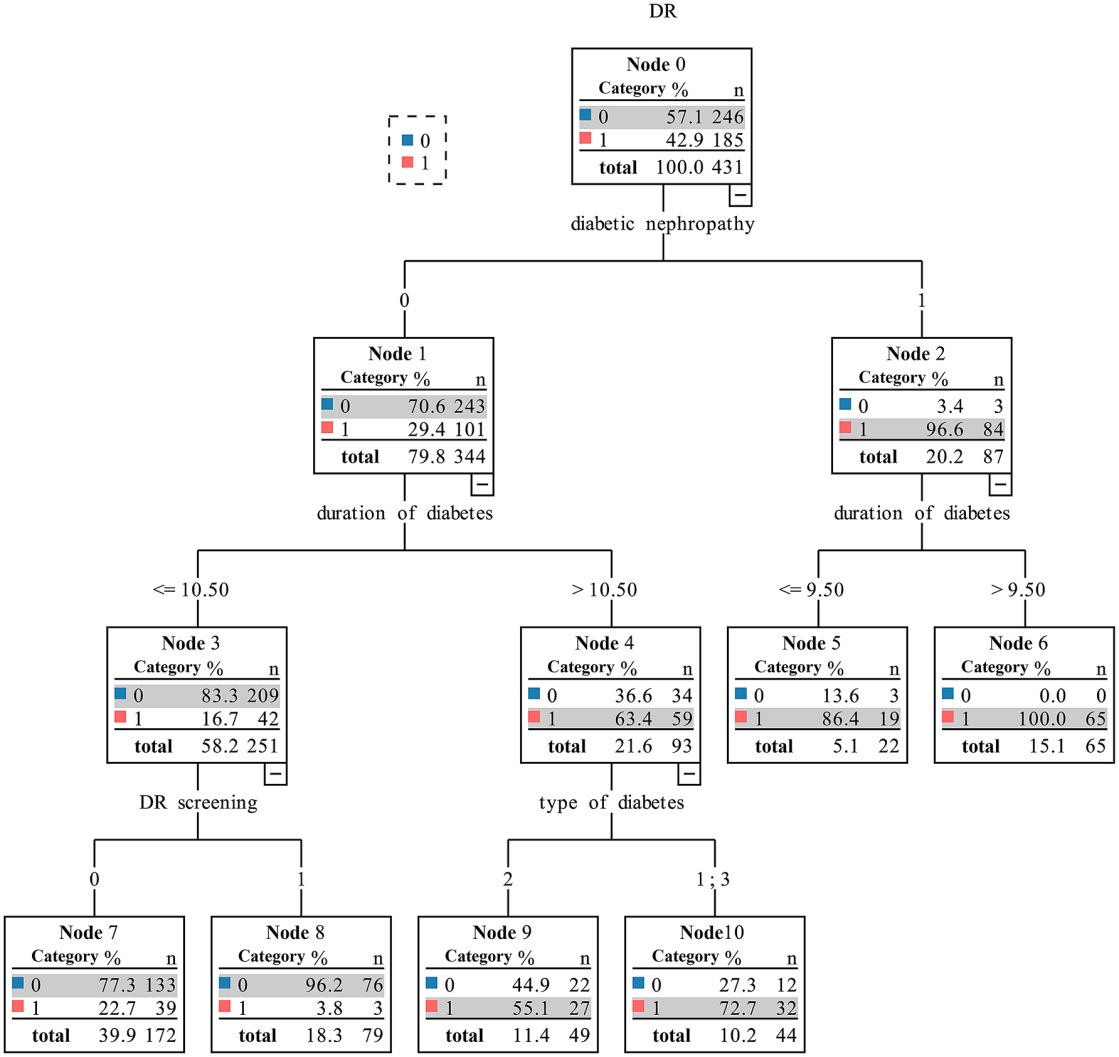
*Decision tree analysis for predicting diabetic retinopathy*. In the figure, the numbers at each node represent the true values, and the categories with a gray background represent outcomes predicted by the model. A blue square of 0 and a red square of 1 represent the absence and presence of diabetic retinopathy, respectively. 431 diabetic patients were included in the analysis, of whom 185 had diabetic retinopathy and 246 did not. Of the patients predicted to be negative for diabetic retinopathy by the model (negative predictive value), 243/344 (70.6%) did not have diabetic retinopathy; of the patient predicted to be positive for diabetic retinopathy by the model (positive predictive value), 84/87 (96.6%) had diabetic retinopathy. The interpretation of the rest of the nodes is similar. With respect to subdivisions according to diabetes type, 1 represents type 1 diabetes mellitus, 2 represents type 2 diabetes mellitus, and 3 represents latent autoimmune diabetes in adults. DR, diabetic retinopathy (color figure online)

**Fig. 2:**
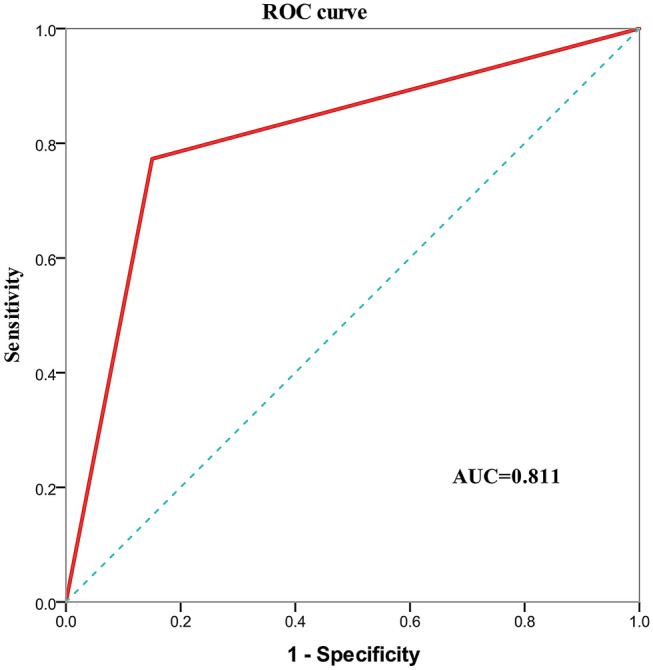
*Receiver-operating characteristic curve of the decision tree model for predicting diabetic retinopathy*. The receiver-operating characteristic curve was generated by calculating the sensitivity and specificity. The Y-axis shows the sensitivity (true positive rate) for predicting diabetic retinopathy. The X-axis shows the 1—specificity (false positive rate) of the diabetic retinopathy prediction model. The red line represents the performance of the decision tree model. The area under the curve for this model is 0.811. ROC, receiver operator characteristic; AUC, area under the curve (color figure online)

To explore whether there were interactions between diabetes type and the risk and protective factors that influenced the occurrence of DR, decision tree analysis was performed. Type of diabetes and three influencing factors (diabetes duration, diabetic nephropathy, and regular DR screening) were included in the decision tree model. In diabetes patients without diabetic nephropathy with a diabetes duration of at least 10.5 years, results showed that those diagnosed with T1DM and LADA had a higher incidence of DR than those with T2DM (72.7% vs. 55.1%). The decision tree is detailed in Fig. 1. The confusion matrix of the decision tree model is shown in Online Resource 2. According to the confusion matrix, the accuracy, specificity, sensitivity, and the F1 value of the model were calculated to be 81.7%, 85%, 77.3%, and 0.78, respectively. The receiver operator characteristic (ROC) curve of the decision tree model is shown in Fig. 2. The model had an area under the curve (AUC) of 0.811 (95% CI: 0.768–0.855), which was considered to indicate good performance.

## Discussion

In this study, age- (± 2 years) and sex-matched patients with T1DM, T2DM, and LADA were enrolled to investigate the relationship between diabetes type and occurrence of DR. DR threatens visual function in 14.77%–22.43% of patients with diabetes in China [12]. The high prevalence of DR in our study population (T1DM 50.5%, T2DM 47.7%, LADA 26.4%) might be explained by the fact that our hospital is one of the top general hospitals in the country, and the conditions of the in patients are complicated. In a study conducted in Korea, the results showed similar prevalence rates of DR in the same three patient populations [13]. However, Lu et al. [14] reported that the DR prevalence was higher in patients with T2DM than in patients with LADA (25% vs. 20.3%, *P* = 0.033). In our study, patients in the LADA group had a shorter diabetes duration and a lower DR prevalence than T1DM and T2DM patients. As seen in the logistic regression analysis, diabetes duration was an independent risk factor for DR. This finding is supported by findings from previous studies [15, 16]. Therefore, the shortest mean diabetes duration in the LADA group was inferred to be related to the lowest DR prevalence seen in this group, as compared to those in the remaining two groups.

Remarkably, LADA patients had higher DR screening rates and underwent the first fundus examination at an earlier time-point than those in the other two groups. The reason for these differences is unclear. Furthermore, in the logistic regression analysis, regular DR screening was an independent protective factor of DR (OR = 0.33, 95% CI: 0.16–0.69, *P* = 0.003). We have not been able to find previous studies that had similar content. The ADA recommends dilated and comprehensive eye examinations every 1–2 years for diabetes patients without evidence of retinopathy, and more frequent examinations are necessary if any level of DR is present or if sight is threatened [17]. However, not every patient undergoes annual dilated and comprehensive eye examinations. In our study, almost half of the diabetes patients never underwent a fundus examination after diagnosis (T1DM 45.5%, T2DM 52.7%, LADA 51.8%). Owing to the preventive effects of therapy and the fact that patients with PDR or macular oedema may be asymptomatic, the ADA emphasized that DR screening should be strongly supported [17]. Additionally, our results suggest that performing screening early and regularly has a positive effect on preventing the occurrence of DR. This may be due to greater awareness of health management in these patients, as well as greater access to physician advice during screening. Given the low DR screening rate, doctors have much work to do.

In the case of diabetic nephropathy, the DR group had worse kidney function and a higher incidence of DN than the NDR group. It has been widely confirmed that DN is associated with DR in diabetes patients [18–22]. Similarly, the current study found that diabetic nephropathy was an independent risk factor of DR. Both the retinas and kidneys are organs supplied by the microvasculature, which is sensitive to fluctuations in blood flow [23]. The progression of retinopathy and nephropathy affects each other, supporting the view of a shared etiological basis and emphasizing the need for a multidisciplinary approach to diabetes care [24].

To investigate whether the occurrence of DR and its related factors are affected by the type of diabetes (T1DM, LADA, and T2DM), diabetes type was included in multivariable logistic regression analysis. We found that diabetes type was not an independent influencing factor of DR. This was consistent with the results of the Fremantle Diabetes Study. In their logistic regression model, diabetes duration, HbA1c, systolic blood pressure, and current smoking were each significantly and independently predictive of retinopathy, but GADA status was not [15]. We further explored whether the type of diabetes affected the occurrence of DR through interaction with other influencing factors. The results of the decision tree analysis indicated that there was an interaction between diabetes type and diabetes duration in patients without nephropathy. In patients with T1DM and LADA with a long disease course, more attention should be paid to health management, and follow-up regarding fundus health should be strengthened.

It has been widely reported that patients with LADA generally have lower triglyceride levels; higher high-density lipoprotein cholesterol levels; and lower BMI, waist-to-hip ratio, and blood pressure than those with T2DM [25]. The prevalence of metabolic syndrome was reported to be significantly higher in T2DM patients than in patients with LADA or T1DM [26]. The baseline analysis of our study revealed similar results. In another study, the inverse association between simultaneous positivity for antibodies against islet cell cytoplasmic antigens (ICA), glutamic acid decarboxylase enzyme (GAD), and tyrosine phosphatase-like transmembrane glycoprotein (IA2) and metabolic syndrome and its components present in LADA patients might imply that LADA patients are phenotypically closer to T1DM patients [27]. In our study, the history of ketoacidosis was similar between LADA and T1DM patients, but it was worse in these two populations than in T2DM patients. LADA patients also had the highest HbA1c levels among the three groups. LADA patients tend to have worse glycaemic control than patients with T2DM [4]. Therefore, it is necessary to reduce the misdiagnosis rate of LADA patients, strictly follow up on their islet function, and formulate an appropriate glycaemic control regimen. Correct diagnosis is the cornerstone of health management and reduction of complications.

A strength of this study was the analysis of influencing factors of DR in patients with LADA, T1DM, and T2DM based on an age- and sex-matched dataset. The interactions between diabetes type and the influencing factors for DR were further explored, which have rarely been reported in previous studies and provide a reference for future research. Admittedly, this study has some limitations. The sample size was not large enough for a more detailed analysis on influencing factors. In addition, this was a retrospective study, and the causal relationship between the variables and DR needs to be explored in future prospective studies and randomized clinical trials with sufficiently long follow-up periods.

In conclusion, this study revealed that the clinical features of LADA are closer to those of T1DM, and patients with LADA present with worse glycaemic control than patients with T2DM. Diabetes type was not an independent influencing factor of DR, but among diabetes patients without diabetic nephropathy with a diabetes duration of at least 10.5 years, T1DM and LADA patients had a higher incidence of DR than those with T2DM. Regular DR screening is an independent protective factor of DR. Correct diagnosis of LADA is necessary, and DR screening needs to be well-implemented.

## Supplementary Information

Below is the link to the electronic supplementary material.

Supplementary file1 (XLSX 10 kb)

Supplementary file2 (XLSX 9 kb)

## Data Availability

The dataset is available from the corresponding author on reasonable request.

## Code availability

The code is available from the corresponding author on reasonable request.
